# Pharmacokinetic and Lipidomic Assessment of the In Vivo Effects of Parishin A-Isorhynchophylline in Rat Migraine Models

**DOI:** 10.1155/2020/9101598

**Published:** 2020-07-06

**Authors:** Chaoqun Zhou, Mingzhen He, Chunyan Peng, Jianjun Yu, Zhifeng Li, Maofu Zhou, Yan Li, Shilin Yang, Hui Ouyang, Yulin Feng

**Affiliations:** ^1^Jiangxi University of Traditional Chinese Medicine, Nanchang, China; ^2^State Key Laboratory of Innovative Drug and Efficient Energy-Saving Pharmaceutical Equipment, Nanchang, China

## Abstract

Migraine is a chronic brain disease that leads to periodic neurological attacks. Parishin A and isorhynchophylline (PI) is the active monomer component extracted from the traditional antimigraine Chinese medicinal combination of Gastrodia and Uncaria, respectively. In this study, using high-performance liquid chromatography coupled with tandem mass spectrometry (HPLC-MS/MS) technology, we performed pharmacokinetic and lipidomic study on migraine model rats after administration of PI. For the detection of the compounds in plasma, AB Sciex Triple Quad™ 4500 was applied for quantitative analysis, and the COSMOSIL C_18_ column (2.1 × 100 mm, 2.6 *μ*m) was used for separation. Isorhynchophylline (ISO: *m*/*z* 384.8–241.2) and its main metabolite rhynchophylline (RHY: *m*/*z* 384.8–160.2) were simultaneously detected under positive ion modes. Besides, parishin A (PA: *m*/*z* 995.1–726.9) and its main metabolite gastrodin (GAS: *m*/*z* 331.1–123.0) were simultaneously detected with negative ion modes. For the analysis of endogenous lipid components, Dionex Ultimate 3000 (UHPLC) Thermo Orbitrap Elite was applied for the detection, and the Waters UPLCRBEH C_18_ column (1.7 *μ*m 100 *∗* 2.1 mm) was used for separation. Chloroform/methanol (2 : 1, *v* : *v*) was used for extraction. The results demonstrated that PI exists significant difference in metabolism between single- and coadministration and can regulate lipid levels associated with migraine.

## 1. Introduction

Migraine causes severe head pain and other disabling neurological symptoms, approximately 14.7% of the global population suffer from migraines, and curative therapies are limited [1, 2]. Although western medicines had achieved certain therapeutic effects, high recurrence rates, toxic side effects, and relatively high costs limit their effectiveness in China [3, 4]. The use of traditional Chinese medicine for the treatment of migraines has been intensely explored. Traditional Chinese medicines provide systemic regulation, have limited side effects, and have multiple targets that minimize drug resistance [5, 6].

Gastrodia-Uncaria (known as Tian Ma and Gou Teng in Chinese) is a commonly used medicinal combination for the treatment of vascular migraines and liver-yang-hyperactivity migraines [7, 8]. In our previous studies, PA and ISO were identified as the major active chemical constituents of Gastrodia and Uncaria and can be absorbed into blood [9]. Besides, further pharmacodynamic assessment of the combination of PA and ISO revealed their antimigraine properties. Because PA and ISO are monomeric components derived from traditional Chinese medicine, the *in vivo* studies of PI will lay a foundation for the formation of a new antimigraine drug with high efficiency, low toxicity, and controllability.

However, the metabolic process of PA and ISO remains unclear, and the advantages of the combination of PI have not been clarified; besides, the efficacy in animal migraine models has not been clarified. Whilst the pathology of migraines remains largely incomplete, a close relationship to lipid metabolism has been demonstrated as altered lipid levels are frequently reported in migraine patients [10–14].

Pharmacokinetic studies provide important references for drug safety and efficacy [15]. Preexperimental data showed that both PA and ISO are unstable in rats and are readily converted to GAS and RHY, respectively. In this study, we employed pharmacokinetic and lipidomic methods to characterize the *in vivo* mechanism of PI from the perspective of drug metabolism and endogenous lipid regulation. Negative ion modes were employed to assess PA and GAS, whilst positive ion modes were used for the assessment of ISO and RHY. Lipidomics were employed to systematically analyze lipid metabolism under PI treatment conditions [16–18].

## 2. Material and Methods

### 2.1. Reference Substance and Chemical Reagents

PA (98%, purity) was purchased from the Purify Technology Development Co., Ltd., Chengdu, China; GAS (98%, purity) was acquired from the Herbpurify Co., Ltd., Chengdu, China; ISO (98.5%, purity) was purchased from the Herbpurify Co., Ltd., Chengdu, China; RHY (98%, purity) was obtained from the Herbpurify Co., Ltd., Chengdu, China; pioglitazone (98%, purity) was supplied by Herbpurify Co., Ltd., Chengdu, China; geniposide (97.6%, purity) was provided by the National Institutes for Food and Drug Control (Beijing, China); plasma was purchased from YiQi Biotechnology Co., Ltd., Henan, China; formic acid, ammonium formate, and isopropanol were purchased from Sigma-Aldrich (St. Louis, MO, USA); methanol, acetonitrile, and formic acid were of chromatographic purity (Fisher, USA).

### 2.2. Animal Handling

Healthy Sprague-Dawley (SD) female rats weighing 180 g ± 20 were provided by Hunan SJA Laboratory Animal Co., Ltd. (animal license number: SCXK (Xiang) 2016-0002). Rats underwent adaptive feeding for one week and were fasted for 12 h. Rats had open access to water throughout the study. For pharmacokinetic analysis, 18 SD rats were randomly divided into 3 groups and 0.4 mL of nitroglycerin (dosage: 10 mg/kg) was injected into the forehead to establish the migraine model [19]. After 1 h, rats were intragastrically administered PA alone (372.4 mg/kg), ISO alone (40 mg/kg), and PI coadministered (ISO: 40 mg/kg, PA: 372.4 mg/kg). For blood sampling, 0.3 mL of blood was taken from the rat orbit prior to drug administration and 0, 5, 15, 30, and 45 min and 1, 2, 4, 6, 8, 10, 12, and 24 h after administration. These blood samples were collected into EP tubes coated with heparin sodium. After centrifugation at 4000 rmp for 10 min, supernatants were collected and stored at −80°C.

For lipidomics, 24 SD rats were randomly divided into three groups: (1) control; (2) model; and (3) administration groups. For the drug-administration groups, 0.4 mL of nitroglycerin was injected into the rat foreheads, and 1 h later, rats were intragastrically administered PI (ISO: 40 mg/kg, PA: 372.4 mg/kg). Blood was collected from the rat eyelids 1 h and 24 h after administration. The collected blood samples were centrifuged at 4000 rmp for 10 min, and supernatants were stored at −80°C.

### 2.3. Preparation of Calibration Standards and Quality Control Samples

For the biological analysis method of simultaneously detecting ISO and RHY, the concentrations of the standard curve was prepared by gradient dilution of the 1 mg/mL mixed storage solution with appropriate amount of acetonitrile solution, followed by adding blank plasma and IS solution to the final concentration of 0.5, 2, 10, 25, 50, 100, and 200 ng/mL. The QC samples included LLOQ (0.5 ng/mL), LOQ (1 ng/mL), MQC (15 ng/mL), and HQC (150 ng/mL); for detecting PA and GAS, the final concentrations (5, 10, 50, 100, 500, 1000, and 2000 ng/mL) of the standard curve were prepared by gradient dilution of the 1.2 mg/mL mixed storage solution with appropriate methanol solution, followed by adding blank plasma and IS solution. The QC samples included LLOQ (5 ng/mL), LOQ (15 ng/mL), MQC (150 ng/mL), and HQC (1500 ng/mL).

For lipidomics, QC samples were prepared by mixing 10 *μ*L of each sample, using PC (17 : 0) at a concentration of 30 *μ*g/mL as the IS.

### 2.4. Sample Processing

For pharmacokinetic studies on RHY and ISO, 50 *μ*L of plasma was transferred into 1.5 mL EP tubes to which 50 *μ*L of pioglitazone hydrochloride (IS: 175 ng/mL) and 250 *μ*L of acetonitrile were added. The mixture was vortexed for 3 min and centrifuged for 10 min at 13000 rpm. 200 *μ*L of supernatants was collected for LC/MS analysis. For GAS and PA assessments, 50 *μ*L of geniposide (IS: 350 ng/mL) and 250 *μ*L of methanol were added to 50 *μ*L of the plasma sample. The mixture was vortexed for 3 min and centrifuged for 10 min at 13000 rpm. 200 *μ*L of supernatants was collected into centrifuge tubes, dried in nitrogen, and dissolved in 200 *μ*L of 5% acetonitrile solution (95 : 5, *v* : *v*).

For lipidomic assessment, 100 *μ*L of serum was accurately absorbed and 300 *μ*L of chloroform/methanol (2 : 1, *v* : *v*, containing IS) was added to each sample. Samples were vortexed for 5 min and subjected to ultrasound for 10 min. Then, the samples were centrifuged at 12000 rpm at 4°C for 10 min. Supernatants were collected and precipitates were extracted in 2 mL of chloroform/methanol (2 : 1, *v* : *v*) twice. All supernatants were dried in nitrogen and dissolved in chloroform/methanol (2 : 1, *v* : *v*). 200 *μ*L of supernatants was transferred to sample vials for detection.

### 2.5. Detection Conditions of LC-MS

For pharmacokinetic studies, the AB Sciex Triple Quad™ 4500 was used for detection. For RHY and ISO chromatographic conditions, the COSMOSIL C_18_ column (2.1 × 100 mm, 2.6 *μ*m) was used at a controlled temperature of 40°C; phase A consisted of 0.1% aqueous formic acid (containing 2.5 mM ammonium acetate), and phase B was acetonitrile. A flow rate of 0.5 mL/min was used with an injection volume of 2 *μ*L. The gradient elution was 0.0–1.2 min, 5% B; 1.2–6.0 min, 5–35% B; 6.0–8.5 min, 35–95% B; 8.5–9.0 min, 5% B; and 9.0–10.0 min, 5% B. Under mass spectrometry conditions, the ESI positive ion mode with multiple reaction monitoring (MRM) was used for detection using the following parameters: ion source temperature = 550°C; ion spray voltage = 4500 V; curtain gas (nitrogen) = 25 psi; atomizing gas (GS 1) = 50 psi; auxiliary gas (GS 2) = 50 psi. Ion pairs were as follows: RHY, *m*/*z* 384.8–160.2 (DP: 122 V; CE: 40 eV); ISO, *m*/*z* 384.8–241.2 (DP: 122 V; CE 40 V). The IS was pyrrolidone hydrochloride, *m*/*z* 357.1–134.1 (DP: 85 V; CE: 35 eV). For GAS and PA, the column temperature was controlled at 40°C; phase A was 0.1% aqueous formic acid, and phase B was acetonitrile; flow rate was 0.5 mL/min; injection volume was 2 *μ*L. The gradient elution was as follows: 0.0–1.2 min, 5% B; 1.2–8.0 min, 5–95% B; 8.0–9.0 min, 95–5% B; 9.0–10.0 min, 5% B; 9.0–10.0 min, 5% B. For mass spectrometry, the ESI negative ion mode with MRM mode detection was employed. Mass spectrometry parameters were as follows: ion source temperature = 550°C; ion spray voltage = −4500 V; curtain gas (nitrogen) = 25 psi; atomizing gas (GS 1) = 50 psi; auxiliary gas (GS 2) = 50 psi. Ion pairs were as follows: GAS, *m*/*z* 331.1–123.0 (DP: −60 V; CE: −20 V); PA, *m*/*z* 995.1–726.9 (DP: −165 V; CE: −39 V). The IS was geniposide, *m*/*z* 433.0–225 (DP: −165 V; CE: −20 eV).

For lipidomics, the Dionex Ultimate 3000 (UHPLC) Thermo Orbitrap Elite was used for LC-MS analysis. The following chromatographic conditions were employed: chromatographic column, Waters UPLCRBEH C_18_ (1.7 *μ*m 100 *∗* 2.1 mm); mobile phase A, aqueous solution with 0.1% formic acid (containing 0.1% 1 mmol/L NH_4_COOH); B, acetonitrile-isopropanol (1 : 1, *v* : *v*) solution containing 0.1% 1 mmol/L NH_4_COOH and 0.1% HCOOH; flow rate, 0.40 m L/min; column temperature, 45°C; injection volume, 4 *μ*L. The optimized gradient elution conditions were as follows: 0–2 min, 35–80% B; 2–9 min, 80–100% B; 9–16 min, 100% B; 16–20 min, 100–35% B. The post time was set as 3 min to balance the system. Mass spectrometry was employed in both positive and negative ion modes. Parameters were optimized as follows in the positive mode: heater temp, 300°C; sheath gas flow rate, 45 arb; aux gas flow rate, 15 arb; sweep gas flow rate, 1 arb; spray voltage, 3.0 kV; capillary temp, 350°C; S-lens RF level, 30%; mass range, *m*/*z* 200–1500. In the negative mode, the parameters were as follows: heater temperature, 300°C; sheath gas flow rate, 45 arb; aux gas flow rate, 15 arb; sweep gas flow rate, 1 arb; spray voltage, 2.5 kV; capillary temp, 350°C; S-lens RF level, 60%; mass range, *m*/*z* 200–1500.

### 2.6. Validation Criteria of Bioanalytical Method

Validation of the pharmacokinetic method was according to the FDA guidelines, including selectivity, linearity, precision, accuracy, matrix effects, extraction recovery, and stability [20].

For the selectivity, the signal-to-noise ratio of the analyte was at least 10. For the linear relationship, the correlation coefficient of the standard curves for each compound was greater than 0.99. For inter- and intraday accuracy (RE) and precision (RSD) of QC, the samples did not exceed 15% and the LLOQ did not exceed 20%. The RSD of mean extraction recoveries, matrix effect, and stability should did not exceed 15%.

### 2.7. Data Analysis

DAS 3.2.8 software was used to analyze the plasma concentrations in the different groups across all experimental time points [21]. Time-concentration curves were drawn using Sigma Plot 10.0. For lipidomics, raw data were converted to the common (mzData) format using Agilent MassHunter Qualitative Analysis B.08.00 software (Agilent Technologies, USA). In the R software platform, the XCMS program was used to assess peak identification times, retention time correction, and automatic integration pretreatments [22]. Data were subjected to IS and weight normalization. Visualization matrices containing the sample name, *m*/*z*-*RT* pair, and peak area were obtained. After removing the ions with a signal-to-noise ratio greater than 500, the ions with the missing values greater than 80% in each group, and the internal and isotopic ions, a total of 1654 features were acquired in the positive mode and 1194 were obtained in the negative mode. After editing, data matrices were imported into SIMCA-P 13.0 (Umetrics, Umea, Sweden), mean-centered, and scaled to Pareto variance. Multivariate analysis was then conducted. Combining the data in positive and negative ion modes, an overview was created using unsupervised principal component analysis (PCA) and a DModX was applied to remove outliers. Supervised orthogonal partial least squares-discriminant analysis (OPLS-DA) was applied to distinguish the contribution of the detected variables to the discrimination between model and control groups [23]. The *R*2 and *Q*2 values were applied to assess the model. Desirable conditions were obtained when the *R*2 was close to 1. Similarly, a *Q*2 value larger than 0.5 indicated good predictability. In addition, the OPLS-DA models were validated by CV-ANOVA. Metabolic pathways were enriched on the MetaboAnalyst website (http://www.metabo-analyst.ca/). IBM SPSS statistics 21 software was used and two-tailed independent Student's *t*-tests were performed to identify markers with a VIP greater than 1. Filtered metabolites (VIP > 1 and *P* < 0.05) were identified as potential biomarkers and matched with the HMDB (http://www.hmdb.ca/), METLIN (https://isometlin.scripps.edu/), MassBank (http://www.massbank.jp/), and ChemSpider (http://www.chemspider.com/). The receiver-operating characteristic (ROC) curve was applied to judge the potential biomarkers in which important biomarkers of migraines had AUC values greater than 0.7 [18].

## 3. Results and Discussion

### 3.1. Optimization of LC-MS/MS Conditions

RHY and ISO are isomers and so different ion pairs were selected to facilitate both the integration and calculation of the sample peak areas. Liquid chromatography conditions were optimized to meet the baseline separation requirements. As both are alkaloids, favorable intensities for mass spectrometry and improved peak conditions were obtained on the C_18_ column. The addition of 0.1% formic acid in the aqueous phase improved the symmetry of the peaks. Acetonitrile promoted sample separation and reduced the column pressure, protecting the ultra high-pressure pumps. Formic acid (0.1%) and acetonitrile were thus used as the mobile phases.

PA and GAS have different structures, so appropriate IS was required. GEN has similar physical, chemical, and structural properties and was selected as the IS. GAS has a relatively large polarity and short peak time, meaning that it was necessary to select a suitable chromatographic column and prolong the analysis time. During sample processing, the double peaks of GAS were eliminated using 5% acetonitrile water (5 : 95/*v* : *v*) to redissolve the GAS and PA. Under acidic conditions, GAS easily formed additive ions with HCOO^−^. When formic acid was used as the mobile phase, 331.1–123.0 was selected for ion pair detection.

Figure S1 shows the representative multiple reaction monitoring (MRM) chromatograms of the determined compounds at 15 minutes after coadministration in migraine model rats. And Figure S2 displays the representative total ion chromatogram (TIC) of plasma samples in positive and negative ion modes. Both of them show the good results of conditional optimization.

### 3.2. Validation of Bioanalytical Method

The standard curve (Figure S3) shows the detected compounds and the chromatogram. The selective study of the method validation indicated good linear relationship and selectivity.  Table S1 shows the intra‐ and interday precision and accuracy of PA, GAS, ISO, and RHY.  Table S2 shows the extraction recovery, matrix effect, and stability (including short-term, long-term, and repeated freeze-thaw conditions) of each compound. The results indicated that the intra‐ and interday accuracy of each compound was between 0.08% and 7.08%, and the precision ranged from 1.50% to 8.39%, indicating high accuracy and precision. The extraction recovery was between 86.18% and 104.12%, and the matrix effect was between 85.82% and 106.83%, indicating good recovery and no effects of the matrix. The RSD of the stability was between 1.04% and 11.36%, which satisfied the analytical requirements. Thus, the conditions met the requirements for PI analysis in the rat plasma.

### 3.3. Pharmacokinetic Study

**Figure 1:**
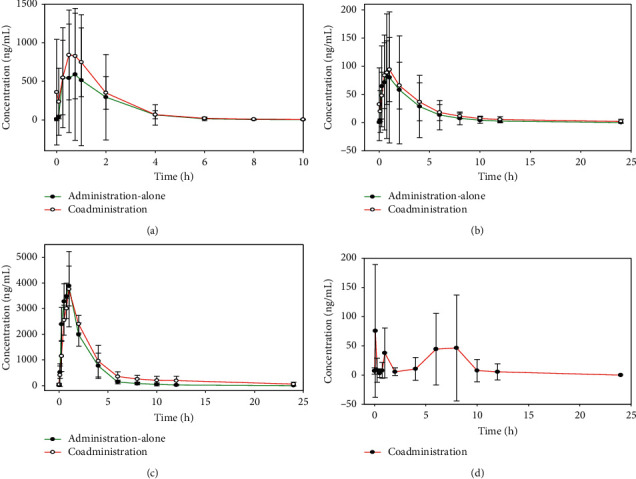
Comparison of plasma concentration-time curves of each compound by administration-alone and coadministration (a) for ISO, (b) for RHY, (c) for GAS, and (d) for PA.

Figure 1 shows the plasma concentration-time curves following single- and coadministration of each compound. The coadministration of ISO, RHY, and GSA was above those of the single-administration groups, whilst PA was almost undetected in either group. Both PA and ISO are unstable in plasma, PA is easily metabolized to GAS, and ISO is easily converted to RHY, resulting in a larger error in the concentration-time curve of the compounds.  Table 1 shows the pharmacokinetic parameters of each compound. Following coadministration, the AUC of ISO was 1.31-fold higher than the administration-alone group, whilst RHY was 1.44-fold higher. In addition, the C-max and T-max of both ISO and RHY were larger than those of the single-administration group, suggesting that coadministration promoted the absorption of ISO and RHY into the blood. The half-life (*t*1/2*z*) of ISO through coadministration was 1.99-fold higher than the administration-alone group, whilst RHY was 3.46-fold higher. The clearance rate (CL *z*/*F*) of ISO in the administration-alone group was 3.15-fold higher than the coadministration group, whilst RHY was 3.16-fold higher. The mean residence time (MRT) of both groups was higher than those of the single-administration group. This indicated that coadministration prolongs the *t*1/2*z* of ISO and RHY, reduces clearance rates, and increases the average residence time, prolonging drug activity *in vivo*. GAS is a slowly transforming metabolite of PA in the blood [24]. When the pharmacokinetic parameters of GAS were optimized via coadministration, the AUC was 1.31-fold higher than the single-administration group, whilst the *t*1/2*z* was 2.79-fold higher and the MRT was 1.84-fold higher. This suggested that coadministration promotes drug efficacy *in vivo*. To date, traditional Chinese medicinal pharmacokinetic studies have been based on healthy experimental animals, discounting the physiological and pathological changes that occur during disease. The pharmacokinetic analyses of drugs used in the treatment of diabetes, cerebral ischemia, hepatobiliary, intestinal disorders, and kidney disease significantly differ from the normal physiological state [25–28]. The disease process can lead to pathological states that influence the activity of related enzymes. As such, animal disease models provide more credible guidance for clinical drug use and enhance practical significance.

### 3.4. Multivariate Statistical Analysis

**Figure 2:**
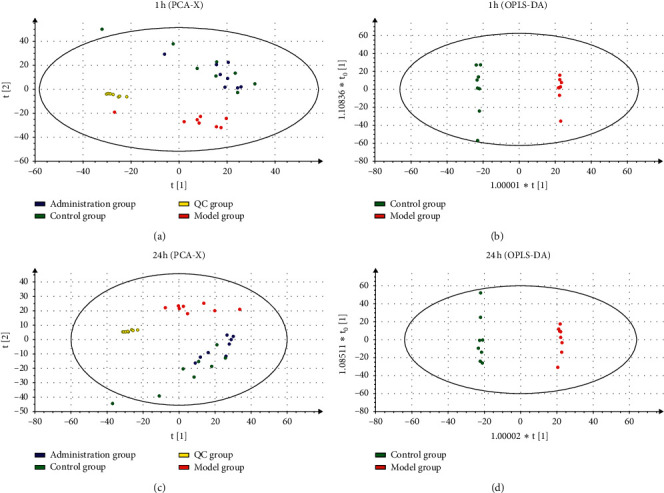
Overview of the sample: (a) PCA score plot in 1 h after modeling; (b) OPLS-DA score plot in 1 h after modeling; (c) PCA score plot in 24 h after modeling; (d) OPLS-DA score plot in 24 h after modeling.

Figure S4 shows a representative total ion chromatogram (TIC) of plasma samples in positive and negative ion modes.  Figure 2 shows the multivariate analysis of potential migraine biomarkers. PCA data are shown in Figure 2(a) (the 1 h group) and Figure 2(c) (the 24 h group). OPLS-DA results are shown in Figure 2(b) (the 1 h group) and Figure 2(d) (the 24 h group). These included the control (green) administration groups at 1 h and 24 h (blue) and model groups at 1 h and 24 h (red). The PCA figures showed that the QC samples displayed good aggregation which reflected the stability of the LC-MS system. The quality of the sample was thus reliable [29]. The PCA analysis was validated no outliers. To further separate the samples and better reflect the differences between control and model groups, samples were analyzed using the supervised mode OPLS-DA, which possesses an improved clustering effect. The results of OPLS-DA showed that the model group and the control group were divided well from each other. Besides, relevant *R*^2^*y* and *Q*^2^*y* values were applied to evaluate the quality of the OPLS-DA model. In the rat lipid metabolism curves, the *R*^2^*y* was 0.966, *R*^2^*x* was 0.861, and *Q*2 was 0.897. In the 24 h group, the *R*^2^*y* was 0.967, *R*^2^*x* was 0.867, and *Q*2 was 0.889. These results indicate that the model displayed improved predictive parameters indicating significant differences in metabolism between the migraine and control groups [30].

### 3.5. Identification of Important Lipid Biomarkers

**Figure 3:**
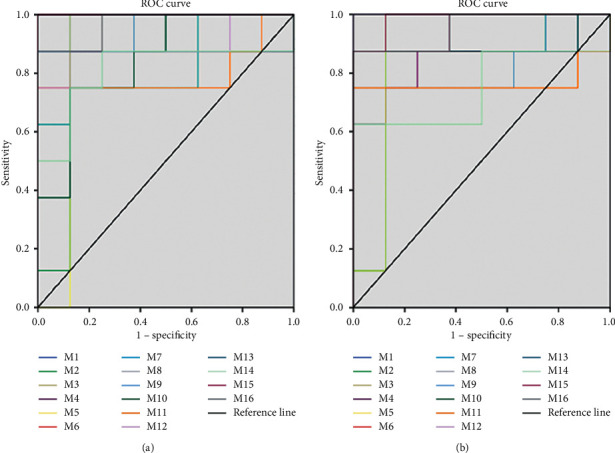
ROC analysis of rat important potential biomarkers: (a)1 h group and (b) 24 h group metabolites.

**Figure 4:**
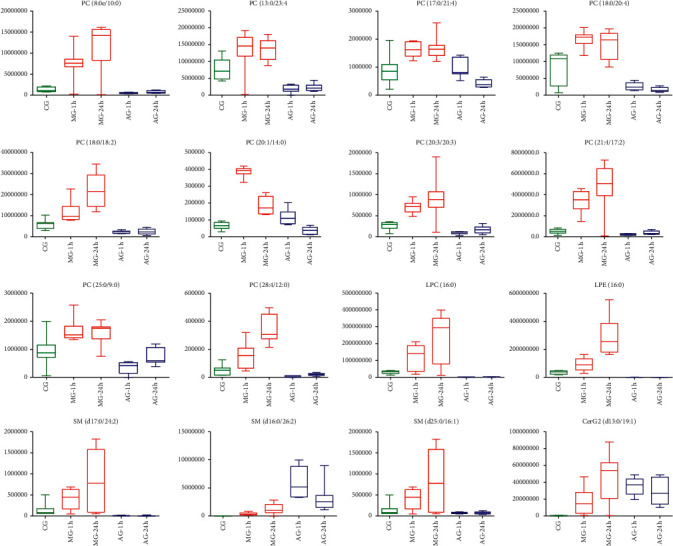
Box plot analysis for important lipid biomarkers by the regulation of PI.

For the assessment of lipid biomarkers, samples were divided into 1 h analysis (including the 1 h control and 1 h model groups) and 24 h analysis groups (including the 24 h control and 24 h model groups). The OPLS-DA model was established to screen contributing variables. A total of 77 variables with a VIP greater than 1 were observed in the 1 h analysis group, and 170 were identified in the 24 h analysis group. The metabolites with a VIP > 1 and *P* < 0.05 were identified as candidates. The accurate mass and its MS/MS fragments of the biomarkers were then identified by matching with the online databases such as HMDB (http://www.hmdb.ca/), METLIN (https://isometlin.scripps.edu/), MassBank (http://www.mass-bank.jp/), and ChemSpider (http://www.chemspider.com/). The error between extraction mass value and experimental mass value was less than 5 ppm. During this stage, 44 and 90 potential lipid biomarkers were identified in the 1 h and 24 h groups, respectively. To assess the importance of each lipid biomarker, the ROC was assessed. A total of 37 biomarkers were obtained according to the AUC values (Figure 3), 8 of which were in the 1 h analysis group, 16 were in the 1 h and 24 h analysis groups, and 13 were in the 24 h analysis group. In the model group, the biomarkers (Table 2) in both 1 h and 24 h groups were considered important. These included phosphatidylcholines (PCs), lysophosphatides (lysoPC and lysoPE), and sphingolipids (Cer and SM). Box plot analysis (Figure 4) of the 16 biomarkers showed a significantly higher content in the model group compared to the control group, followed by a downward trend following PI administration.

### 3.6. Analysis of Metabolic Pathways and Important Lipid Biomarkers

**Figure 5:**
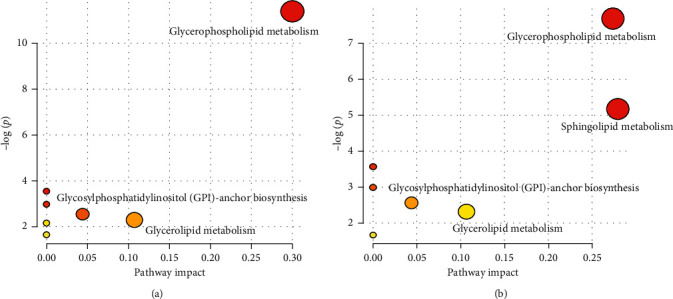
Metabolic pathway analysis: (a) for 1 h group and (b) for 24 h group.

Metabolic pathway analysis is shown in Figure 5.  Figure 5(a) provides the metabolic network of migraines of the 1 h analysis group. These include glycerophospholipid, glycerolipid, and glycosylphosphatidylinositol (GPI)-anchor biosynthesis metabolism.  Figure 5(b) shows the metabolic network of migraines of the 24 h analysis group. These include sphingolipids, linoleic acid, and glycerolipid metabolism. According to the impact value, glycerophospholipid and sphingolipid metabolism were the major pathways of migraine regulation. PC, LPE, and LPC were involved in glycerophospholipid metabolism. In addition, SM and ceramides participated in sphingolipid metabolism. Glycerophosphatidyl is the most abundant type of phospholipids in the body with the basic structures of phosphatidic acid and substituents, which can be divided into phosphatidylcholine, phosphatidylethanolamine, phosphatidylserine, phosphatidylglycerol, phosphatidylinositol, and so on, according to different substituents [31, 32]. The main function is to form biofilm and participate in protein recognition and signal transduction in cell membranes [33]. Lysophospholipids are produced by the hydrolysis of glycerophospholipids or sphingomyelins to remove one fatty acid side chain [34]. Among which, lysoPC and lysoPE have high abundance and possess important physiological functions and activities [35]. In this study, PC components, LPC (16 : 0), and LPE (16 : 0) were identified as important lipid biomarkers of migraine, which involved in the metabolic pathway of glycerophospholipids. Compared with the blank group, the content in migraine model rats increased significantly. PC is the precursor of acetylcholine and can be hydrolyzed into choline in the body [36]. Choline enters the brain along with the blood circulatory system and combines with acetic acid to convert to acetylcholine. Acetylcholine is an important neurotransmitter that promotes communication between brain nerve cells, especially brain neurons [37, 38]. When the content is increased, the information transmission speed between the brain neurons is accelerated, and the brain nerve function is enhanced. In migraine, promoting the production of PC may regulate the activity of brain tissue. Besides, studies have shown that most migraine sufferers suffer from anxiety and insomnia with or without other stimuli, and these causes of migraine can lead to oxidative stress; when oxidative stress occurs, the production of free radicals activates phospholipase A2 (a biological enzyme that promotes the production of lysophospholipids), thereby increasing the content of lysophospholipids [39–42].

Sphingolipids are the main components of eukaryotic cell membranes, which play an important role in regulating the fluidity of lipid bilayer membranes and in cell signal transduction [43]. Sphingolipids mainly exist in the nervous system, including sphingosine, ceramide (Cer), and sphingomyelin (SM). Human research demonstrated that changes in the balance of sphingolipid metabolism were closely related to neurological diseases [44, 45]. Another basic scientific study suggested that sphingolipids may be involved in pain-related neurological and signaling pathways [46–48]. In this study, we found SM (d17 : 0/24 : 2), SM (d16 : 0/26 : 2), SM (d25 : 0/16 : 1), and CerG2 (d13 : 0/19 : 1) were important markers for migraine.

## 4. Conclusions

In this study, the metabolic processes of PI in migraine models were assessed using a combination of pharmacokinetic and lipidomic methods *in vivo* (endogenous lipid components) and *in vitro* (PI components). Through comparison of the pharmacokinetic parameters following co- or single-administration, the advantages of coadministration were highlighted. In addition, important lipid biomarkers of migraines were revealed through lipidomic analysis, and the regulatory effects of PI on migraines were confirmed. These studies provide practical reference values for the clinical application of PI.

## Figures and Tables

**Table 1:** Pharmacokinetic parameters of the determining compounds.

Parameters	Unit	ISO		RHY		GAS		PA	
		Single	Combine	Single	Combine	Single	Combine	Single	Combine
AUC (0 − *t*)	*μ*g/L *∗* h	1340.01	1755.85	313.95	412.82	9934.28	12757.67	0.00	304.22
AUC (0 − ∞)	*μ*g/L *∗* h	1340.09	1768.51	314.00	455.57	9935.51	13028.09	0.00	304.22
MRT (0 − *t*)	h	1.76	1.98	3.21	4.90	2.14	3.95	0.00	4.58
MRT (0 − ∞)	h	1.77	2.34	3.21	8.57	2.15	4.36	0.00	4.58
*t*1/2*z*	h	1.90	3.79	1.63	5.64	1.02	2.85	0.00	0.69
T-max	h	0.54	0.58	0.63	0.96	0.83	1.08	0.00	1.99
*V z*/*F*	L/kg	241.72	174.09	663.02	614.92	56.61	110.10	0.00	1177.20
CL z/*F*	L/h/kg	88.98	28.16	331.66	104.87	38.38	29.90	0.00	8247.98
C-max	*μ*g/L	645.28	867.07	88.35	94.10	4082.17	3853.50	0.00	114.42

**Table 2:** Identification of important potential serum lipidomic biomarkers in migraine model rats.

No.	Lipid biomarkers	Ion mode	M.F	Mass (*M*/*z*)	Rt (min)	FC (control/model)	*P* value	VIP	AUC
						1 h; 24 h	1 h; 24 h	1 h; 24 h	1 h;24 h
1	PC (8 : 0*e*/10 : 0)	[M + HCOO]^−^	C_27_ H_55_ O_9_ N_1_ P_1_	568.3620	4.60	0.18; 0.11	4.37*E* − 04; 1.12*E* − 04	10.848; 9.945	0.88; 0.88
2	PC (25 : 0/9 : 0)	[M + HCOO]^−^	C_43_ H_85_ O_10_ N_1_ P_1_	806.5917	13.04	0.57; 0.59	9.82*E* − 03; 1.51*E* − 02	3.330; 2.141	0.89; 0.81
3	PC (20 : 1/14 : 0)	[M + HCOO]^−^	C_43_ H_83_ O_10_ N_1_ P_1_	804.5760	11.49	0.51; 0.41	1.96*E* − 04; 2.12*E* − 02	1.937; 1.369	1.00; 0.78
4	PC (13 : 0/23 : 4)	[M + HCOO]^−^	C_45_ H_81_ O_10_ N_1_ P_1_	826.5604	9.80	0.58; 0.58	3.34*E* − 02; 2.94*E* − 03	8.614; 6.792	0.86; 0.91
5	PC (17 : 0/21 : 4)	[M + HCOO]^−^	C_47_ H_85_ O_10_ N_1_ P_1_	854.5917	11.74	0.55; 0.53	3.20*E* − 03; 4.64*E* − 03	3.534; 2.470	0.88; 0.89
6	PC (21 : 4/17 : 2)	[M + HCOO]^−^	C_47_ H_81_ O_10_ N_1_ P_1_	850.5604	9.34	0.15; 0.11	2.19*E* − 06; 9.69*E* − 05	8.038; 6.412	1.00; 0.88
7	PC (28 : 4/12 : 0)	[M + HCOO]^−^	C_49_ H_89_ O_10_ N_1_ P_1_	882.6230	12.88	0.33; 0.15	9.93*E* − 03; 2.53*E* − 06	1.265; 1.736	0.83; 1.00
8	PC (18 : 0/18 : 2)	[M + H]+	C_44_ H_85_ O_8_ N_1_ P_1_	786.6007	11.94	0.5; 0.27	9.35*E* − 03; 9.98*E* − 05	3.445; 5.129	0.92; 1.00
9	PC (18 : 0/20 : 4)	[M + H]+	C_46_ H_85_ O_8_ N_1_ P_1_	810.6007	11.77	0.62; 0.4	2.30*E* − 02; 9.21*E* − 06	2.354; 3.620	0.84; 0.98
10	PC (20 : 3/20 : 3)	[M + H]+	C_48_ H_85_ O_8_ N_1_ P_1_	834.6007	11.08	0.25; 0.13	4.73*E* − 03; 1.11*E* − 03	4.455; 5.632	0.80; 0.77
11	LPC (16 : 0)	[M + H]+	C_24_ H_51_ O_7_ N_1_ P_1_	496.3398	4.02	0.39; 0.12	4.48*E* − 03; 1.15*E* − 04	1.101; 2.053	0.88; 1.00
12	LPE (16 : 0)	[M − H]^−^	C_23_ H_47_ O_7_ N_1_ P_1_	480.3096	2.98	0.64; 0.55	1.43*E* − 02; 2.29*E* − 03	6.555; 5.865	0.89; 0.92
13	SM (d25 : 0/16 : 1)	[M + HCOO]^−^	C_47_ H_94_ O_8_ N_2_ P_1_	845.6753	15.50	0.36; 0.18	2.59*E* − 02; 2.22*E* − 02	1.880; 2.112	0.81; 0.75
14	SM (d17 : 0/24 : 2)	[M + CH3COO]^−^	C_48_ H_94_ O_8_ N_2_ P_1_	813.6844	14.42	0.03; 0.01	2.90*E* − 04; 2.28*E* − 03	2.678; 3.152	1.00; 0.98
15	SM (d16 : 0/26 : 2)	[M + H]^+^	C_47_ H_94_ O_6_ N_2_ P_1_	857.6753	13.97	0.03; 0.01	1.27*E* − 02; 3.49*E* − 04	1.768; 2.711	0.97; 0.95
16	CerG2 (d13 : 0/19 : 1)	[M + H]^+^	C_44_ H_84_ O_13_ N_1_	834.5937	11.08	0.32; 0.18	1.54*E* − 03; 1.98*E* − 03	4.688; 5.277	0.88; 0.77

## Data Availability

The data used to support the findings of this study are included within the article.

## Abbreviations

PI: Parishin A and isorhynchophylline
HPLC-MS/MS: High-performance liquid chromatography coupled with tandem mass spectrometry
ISO: Isorhynchophylline
RHY: Rhynchophylline
PA: Parishin A
GAS: Gastrodin
MRM: Multiple reaction monitoring
SD: Sprague-Dawley
PCA: Principal component analysis
OPLS-DA: Orthogonal partial least squares-discriminant analysis
ROC: Receiver-operating characteristic
TIC: Total ion chromatogram
CL: Clearance rate
MRT: Mean residence time.

## Supplementary Materials

Supplementary Materials
*Figure S1*. Representive MRM chromatograms of the determined compounds at 15 minutes after coadministration in migraine model rats. *Figure S2*. The representative total ion chromatogram (TIC) of plasma samples in positive and negative ion modes for the pharmacokinetic study. *Figure S3*. Calibration curves of isorhynchophylline, rhynchophylline, parishin A, and gastrodin in plasma samples. *Table S1*. Inter‐ and intraday precision and accuracy for the determination of compounds. *Table S2*. Extraction recovery, matrix effect, and stability study for the determination of the compounds.
