# Supplementary material for: Pharmacokinetic and Lipidomic Assessment of the In Vivo Effects of Parishin A-Isorhynchophylline in Rat Migraine Models
Source: J Anal Methods Chem. 2020 Jul 6;2020:9101598. doi: 10.1155/2020/9101598 (PMC7362284; doi:10.1155/2020/9101598)
Supplement: Supplementary Materials — Figure S1. Representive MRM chromatograms of the determined compounds at 15 minutes after coadministration in migraine model rats. Figure S2. The representative total ion chromatogram (TIC) of plasma samples in positive and negative ion modes for the pharmacokinetic study. Figure S3. Calibration curves of isorhynchophylline, rhynchophylline, parishin A, and gastrodin in plasma samples. Table S1. Inter‐ and intraday precision and accuracy for the determination of compounds. Table S2. Extraction recovery, matrix effect, and stability study for the determination of the compounds. [file 9101598.f1.pdf]

Supplementary materials

Figure S1. Representive MRM chromatograms of the determined compounds at 15 minutes after co-administration in migraine model rats.

Figure S2. The representative total ion chromatogram (TIC) of plasma samples in positive and negative ion mode for pharmacokinetics study.

Figure S3. Calibration curves of isorhynchophylline, rhynchophylline, parishin A and gastrodin in plasma samples

Figure S4. The representative total ion chromatogram (TIC) of plasma samples in positive and negative ion mode for lipidomics study.

Table S1. Inter- and intra-day precision, accuracy for the determining of compounds.

Table S2. Extraction recovery, matrix effect and Stability study for the determining of the compounds.

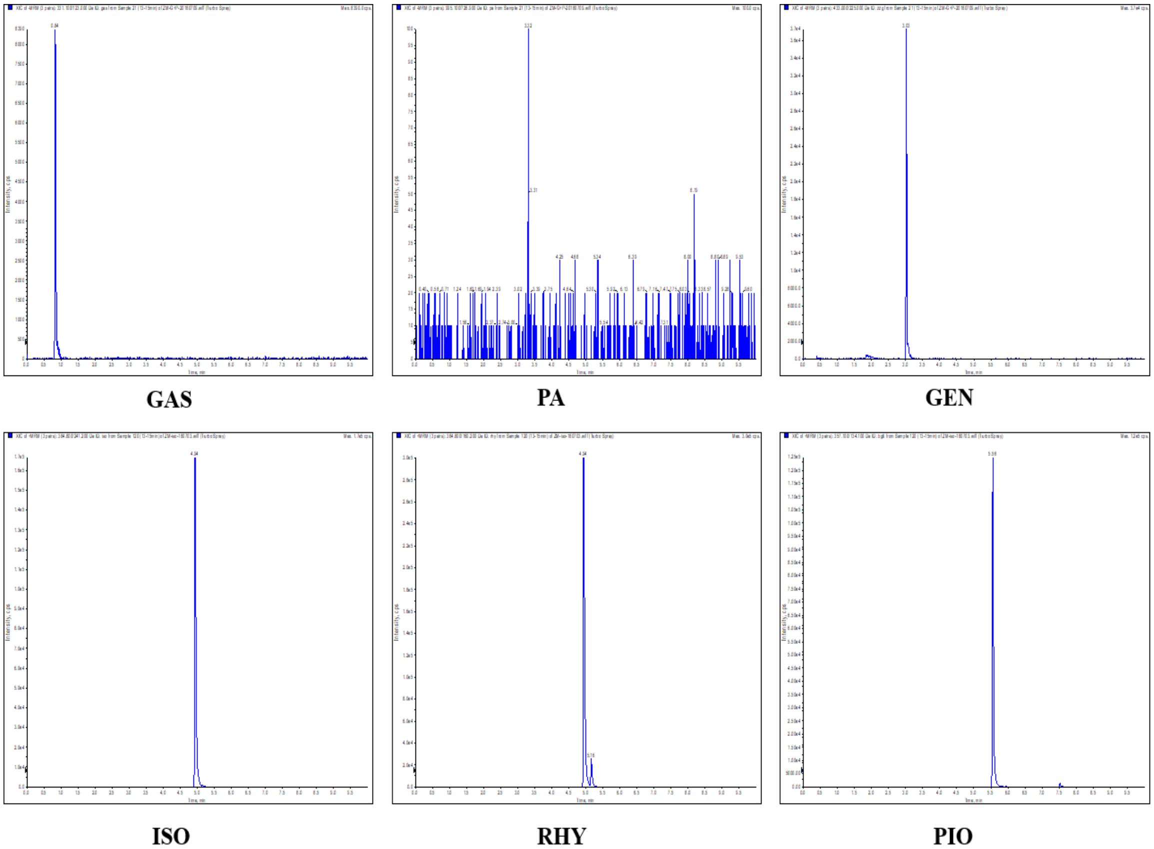

Figure S1. Representive MRM chromatograms of the determined compounds at 15 minutes after co-administration in migraine model rats.

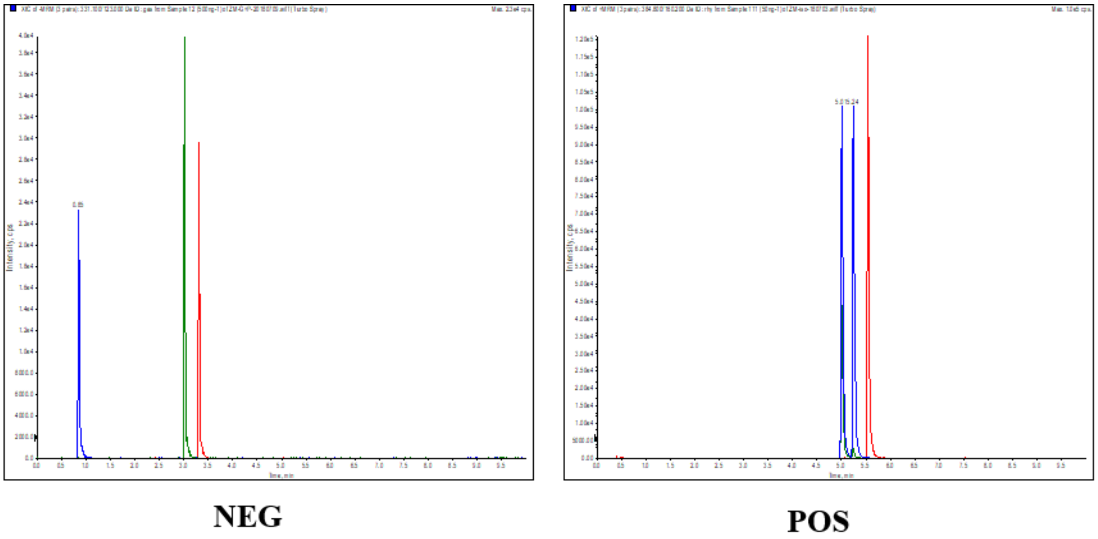

Figure S2. The representative total ion chromatogram (TIC) of plasma samples in positive and negative ion mode for pharmacokinetics study.

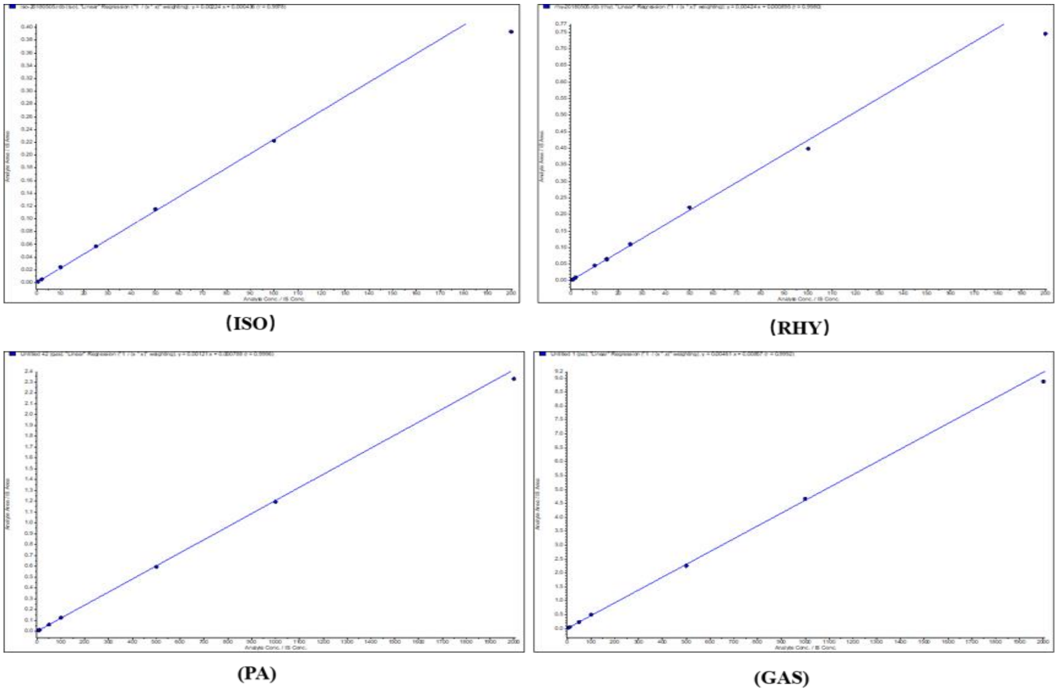

Figure S3. Calibration curves of ISO, RHY, PA and GAS in plasma samples

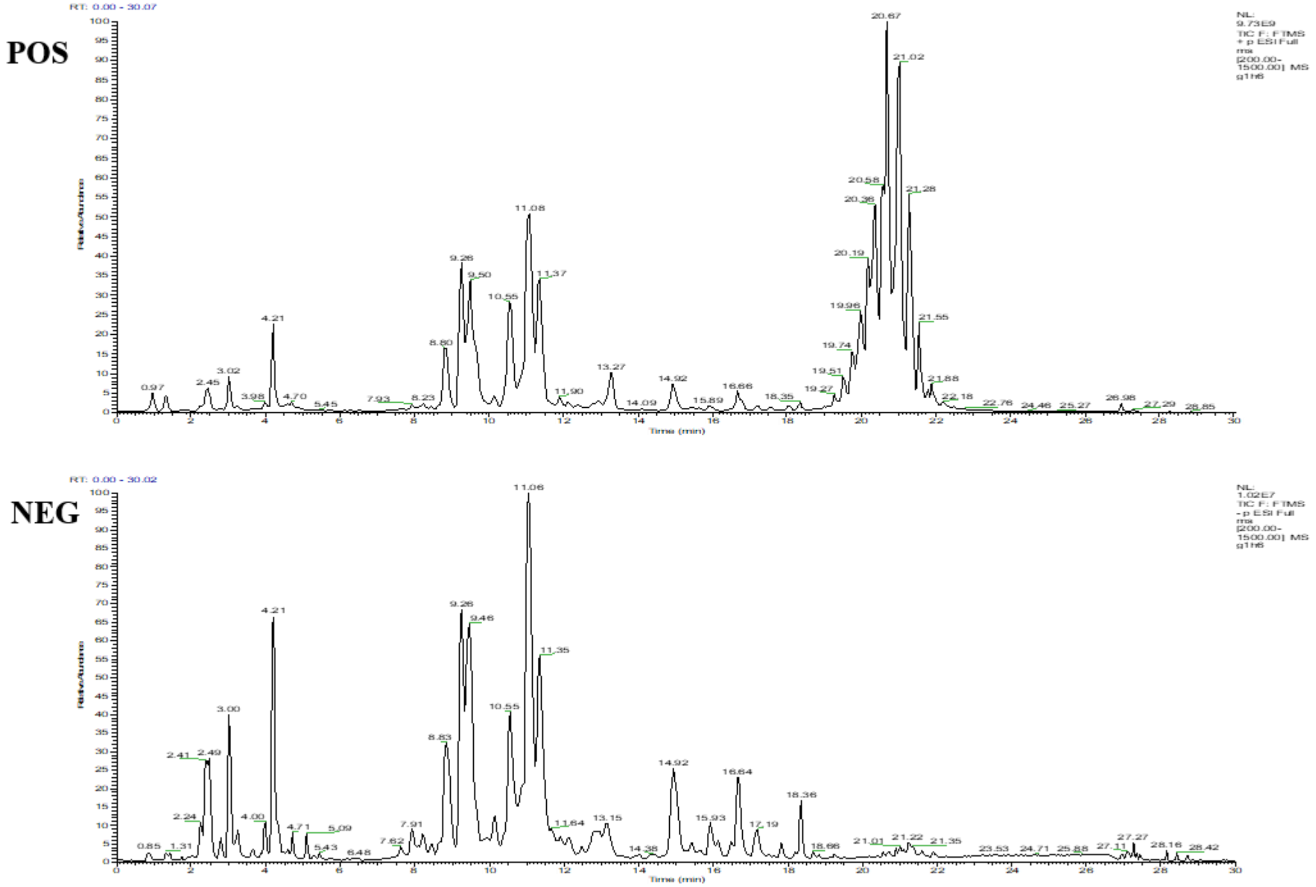

Figure S4. The representative total ion chromatogram (TIC) of plasma samples in positive and negative ion mode for lipidomics study.

Table S1. Inter- and intra-day precision, accuracy for the determining of compounds.

Compounds	Prepared conc (ng / m L)	Inter-batch（n=5）			Intra-batch（n=5）		
		Measured conc (Mean ±SD, ng / m L)	Accuracy (RE, %)	Precision (RSD, %)	Measured conc ((Mean ±SD ng / m L)	Accuracy (RE, %)	Precision (RSD, %)
PA	5	4.90±0.31	2.10	4.98	4.93±0.25	1.24	2.53
	15	14.34±0.70	4.44	4.53	13.94±0.83	7.08	3.04
	150	159.80±5.07	6.60	4.02	157.80±3.42	5.20	6.87
	1500	1520.00±76.49	1.12	5.09	1512.00±50.70	0.86	3.01
GAS	5	4.99±0.33	0.08	3.06	5.05±0.33	1.10	4.22
	15	14.02±0.70	6.56	5.63	14.04±0.77	6.36	4.67
	150	155.00±2.55	3.83	3.28	152.00±4.74	1.28	6.71
	1500	1586.00±40.99	5.60	2.24	1574.00±87.92	4.84	2.50
ISO	0.5	0.50±0.03	0.42	6.02	0.51±0.03	2.64	6.47
	1	0.97±0.08	3.17	7.29	0.99±0.03	0.78	2.62
	15	16.60±0.60	3.82	3.52	14.60±0.30	2.70	2.24
	150	142.33±4.03	5.20	7.25	146.00±1.22	2.76	2.21
RHY	0.5	0.50±0.03	0.33	6.00	0.50±0.02	0.09	8.38
	1	0.99±0.04	0.44	3.81	1.03±0.04	2.88	6.23
	15	15.75±0.49	5.00	3.11	15.42±0.16	2.80	1.50
	150	141.17±4.22	5.20	8.39	141.17±3.87	6.50	2.74

Table S2. Extraction recovery, matrix effect and Stability study for the determining of the compounds.

Com- pounds	Prepared conc(ng/mL)	Extraction recovery (n=6)		Matrix effect(n=6)		Stability study（(n=6, Mean ±SD）					
		Measured conc (Mean ±SD)	RSD（%）	Measured conc (Mean ± SD)	RSD（%）	Short-term stability (Mean ±SD)	RSD (%)	Long-term stability (Mean ±SD)	RSD (%)	Freeze-thaw stability (Mean ±SD)	RSD (%)
PA	15	13.55±0.97	7.17	15.35±1.31	8.59	15.26±0.93	5.95	14.21±0.83	5.84	13.35±0.5	3.75
	150	150.62±5.78	3.83	151.66±2.60	1.71	137.00±2.82	2.06	145.00±1.52	1.04	152.00±2.8	1.84
	1500	1467.60±80.70	5.50	1602.45±68.07	4.29	1543.00±50.25	3.26	1499.00±63.21	4.21	1445.00±70.97	4.91
GAS	15	13.74±0.71	5.14	14.84±0.79	5.31	13.82±0.21	1.52	15.46±0.61	3.95	14.28±0.31	2.17
	150	150.30±4.28	2.85	146.24±5.28	3.61	132.16±10.20	7.70	140.00±4.83	3.45	155.00±7.28	4.70
	1500	1508.40±34.5	2.30	1589.55±42.30	2.66	1435.00±30.38	2.11	1556.00±50.50	3.25	1450.00±77.17	5.32
ISO	1	0.91±0.04	4.33	1.06±0.01	11.52	0.93±0.03	3.23	0.88±0.01	11.36	0.90±0.05	5.55
	15	15.03±0.78	5.17	15.66±0.77	4.91	15.12±1.00	6.61	14.23±0.67	4.71	14.83±1.32	8.90
	150	153.74±6.12	3.98	155.54±5.82	3.74	147.89±3.83	2.59	138.25±2.65	1.91	155.65±5.00	3.21
RHY	1	0.97±0.07	6.98	0.90±0.09	9.95	1.01±0.04	3.96	1.06±0.05	4.72	0.98±0.06	6.12
	15	14.63±0.43	2.93	15.70±0.87	5.56	13.38±1.51	11.28	14.16±1.20	8.47	13.53±0.8	5.91
	150	154.65±4.94	3.18	154.32±7.09	4.60	155.83±6.12	3.92	134.28±3.16	2.35	145.55±5.12	3.52
